# Catheters with Dual-Antimicrobial Properties by Gamma Radiation-Induced Grafting

**DOI:** 10.3390/pharmaceutics15030960

**Published:** 2023-03-16

**Authors:** Lorena Duarte-Peña, Héctor Magaña, Emilio Bucio

**Affiliations:** 1 Departamento de Química de Radiaciones y Radioquímica, Instituto de Ciencias Nucleares, Universidad Nacional Autónoma de México, Circuito Exterior, Ciudad Universitaria, Ciudad de Mexico 04510, Mexico; 2 Facultad de Ciencias Químicas e Ingeniería, Universidad Autónoma de Baja California, Calzada Universidad 14418, Parque Industrial Internacional Tijuana, Tijuana 22390, Mexico

**Keywords:** antimicrobial, antifouling, drug delivery, pH sensitivity, zwitterionic polymers, gamma radiation

## Abstract

Dual antimicrobial materials that have a combination of antimicrobial and antifouling properties were developed. They were developed through modification using gamma radiation of poly (vinyl chloride) (PVC) catheters with 4-vinyl pyridine (4VP) and subsequent functionalization with 1,3-propane sultone (PS). These materials were characterized by infrared spectroscopy, thermogravimetric analysis, swelling tests, and contact angle to determine their surface characteristics. In addition, the capacity of the materials to deliver ciprofloxacin, inhibit bacterial growth, decrease bacterial and protein adhesion, and stimulate cell growth were evaluated. These materials have potential applications in the manufacturing of medical devices with antimicrobial properties, which can reinforce prophylactic potential or even help treat infections, through localized delivery systems for antibiotics.

## 1. Introduction

The biocontamination of both urinary and central line catheters is one of the principal causes of nosocomial infections, mainly in patients who are in intensive care units [1,2]. One of the reasons for this is that these devices are made of polymeric materials that have a tendency towards microorganism contamination in biological environments. According to surveys carried out in different countries, it is estimated that one in seven hospitalizations presents an incidence of nosocomial infection, of which approximately 25% are associated with the use of medical devices [3,4,5]. The National Healthcare Safety Network (NHSN) reports eight types of microorganisms that cause the most nosocomial infections, among which, the following are prominent: *Staphylococcus aureus*, *Escherichia coli*, and coagulase-negative *staphylococci* [6].

Therefore, searching for materials that are resistant to bacterial contamination is relevant to the medical field [7,8,9]. A material can present resistance to contamination by microorganisms through two mechanisms [10]. The first mechanism consists of the incorporation of active agents into the material; it can be in its internal structure as groups of quaternary amines [11,12] or stored to be released at a site of interest. Among the most widely used active agents for release are antibiotics and silver or zinc metallic nanoparticles with antibacterial properties [13,14,15]. The second mechanism is based on the generation of materials whose surface prevents the adhesion of the microorganism and its proliferation. These materials generally owe their antifouling capacity to the formation of superficial hydration layers stabilized by van der Waals interactions or electrostatic interactions, as in the case of zwitterionic polymers. The development of materials with dual antimicrobial capacity, that is, materials capable of both preventing adhesion and releasing an active agent, is a challenge for materials science [16,17].

Modified systems for the release of active agents are a point of interest in biomedicine because they can provide the optimal amount of drug at the right time and place, giving rise to a continuous release of therapeutic dose without reaching maximum levels, thus avoiding side effects caused by large drug discharges and concentrating the drug in the affected area [18,19]. Within these systems are smart polymers, that is, polymers that respond to external stimuli, such as temperature, pH, ionic strength, or light, by changing their structure, which allows more control over the load and release of active agents depending on the environmental conditions [20]. The poly 4-vinylpyridine (P4VP) is pH-sensitive polymer that undergoes protonation at a pH below its pK_a_. This leads to the formation of cations that repel each other thus increasing the distance between the chains of the material and changing its structure.

Zwitterionic polymers have a high antifouling capacity because their ion distribution allows them to create an electrostatically stabilized surface hydration layer, which significantly reduces bacterial adhesion to these surfaces, in addition to being highly hydrophilic systems [21,22,23]. However, the synthesis of these materials is limited by the low solubility of the polymer in the solvents commonly used for polymerization. Due to this, alternative techniques are used to obtain these materials, such as the functionalization of ionic polymers with an ion of opposite charge, to form the zwitterion in situ [24]. Hydrogels developed with this type of polymer have shown relevant antifouling properties and good biocompatibility [25,26,27,28].

This work presents the development of PVC catheters modified with a 4-vinylpyrinidine and zwitterionic polymer to provide their surface with antifouling capacity and pH sensitivity. This material constitutes a dual antimicrobial system that has the ability to load and release ciprofloxacin. This system allows the localized release of the antibiotic because the drug is stored in the device, which can help improve drug efficiency. The modification was carried out by graft polymerization of 4VP using gamma radiation as the initiator and subsequently a zwitterion was formed by the functionalizing of the grafted 4VP with 1,3-propane sultone (PS). The synthesized materials were characterized to determine their antimicrobial capacity. Materials with dual antimicrobial capacity have potential applications in the manufacturing of medical devices that can reinforce their prophylactic potential or even help treat infections. In this case, modified catheters represent an alternative device which can reduce the nosocomial infections associated with traditional catheter use.

## 2. Materials and Methods

### 2.1. Materials

PVC catheters (outer diameter 3 mm and thickness 0.5 mm) were from Biçakcilar (Istanbul, Turkey). 4VP (95%), 1,3-propane sultone (PS), and dimethylformamide anhydrous were purchased from Aldrich Chemical, Saint Louis, MO, USA. 4VP was purified by vacuum distillation to remove the inhibitor. Chloride (NaCl), potassium chloride (KCl), sodium phosphate dibasic (NaH_2_PO_4_), and potassium phosphate monobasic (KHPO_4_) were also purchased from Aldrich Chemical, Saint Louis, MO, USA; these materials were used as received. Ciprofloxacin was from Sigma Aldrich. Distillate water was used for all the assays. Software DDSolver from Excel was used for modeling drug delivery. The gamma-ray source was a ^60^Co Gammabeam 651-PT of Nordion International Inc from Ottawa, ON, Canada proportioned by the Nuclear Science Institute at Universidad Nacional Autónoma de México (ICN-UNAM).

### 2.2. Synthesis of PVC-g-4VP

The 4VP graft on PVC was performed using the direct irradiation method, following the parameters used in previous studies to obtain graft percentages of 12 and 23%. A sample of PVC approximately 6 cm in length was placed in a glass ampoule, a solution of 4VP in H_2_O/MeOH was added, and oxygen was removed by air displacement with argon bubbling for 15 min. The sealed ampoule was kept at 5 °C for 4 h and irradiated using gamma radiation. The grafted catheters were removed and cleaned with methanol. Finally, the samples were dried for 12 h at 30 °C in a vacuum oven, and the percentage of grafting was calculated by the difference in weight using Equation (1), where W_f_ is the weight of the grafted sample (g) and W_i_ is the weight of the sample without modification (g).

Grafting (%) = (W_f_ − W_i_) × 100/W_i_ (1)

### 2.3. Formation of PVC-g-4VP/4VPPS Graft by Functionalization

A dry and weighed sample of PVC-*g*-4VP was placed in a glass ampoule and left under vacuum for 20 min. Then, a solution of PS in dimethylformamide anhydrous was added, the ampoule was sealed, and the solution was heated for a certain period of time. Finally, the modified material was removed, washed with methanol and water for 12 h, and dried at 30 °C under a vacuum for 12 h. PS reacts quickly with water, hydrolyzing to hydroxysulfonic acid, so the reaction must be carried out under anhydrous conditions. The reaction yield was calculated using Equation (2), where M_f_ is the final weight of the material, M_i_ is the initial weight of the material, and 4VP (%) is the percentage of 4VP grafting in the initial material.

Reaction yield (%) = (M_f_ − M_i_) × 8607.7)/(M_i_ × 4VP(%)) (2)

The effect of the different reaction conditions was studied, varying the temperature (50, 60, and 70 °C), the reaction time (30, 45, 60, and 75 min), and the concentration of PS (0.35, 0.5, 0.65, 0.8, 0.8, and 1 M).

### 2.4. Infrared Spectroscopy and Thermal Analysis

Infrared spectroscopy was performed on a Perkin Elmer Spectrum 100 spectrophotometer (Perkin Elmer Cetus Instruments, Norwalk, CT, USA) with 16 scans, in the ATR module, in the range of 4000 to 650 cm^−1^. On the other hand, the thermal behavior was monitored by TGA under a nitrogen atmosphere from 30 to 700 °C at a heating rate of 10 °C/min using a TGA Q50 (TA Instruments, New Castle, DE, USA).

### 2.5. Swelling and Contact Angle

For the swelling tests, a dry sample was weighed and placed in a glass with distilled water at 25 °C. Once removed from the beaker, excess solvent was removed from the sample and it was weighed every 5 min for the first 15 min and then at 0.5, 1, 2, 4, 6, and 12 h. The swelling percentage was determined using Equation (3), where W_2_ is the weight of the swollen sample and W_1_ is the dry sample weight.

Swelling (%) = (W_2_ − W_1_) × 100/W_1_ (3)

The contact angle provided information on the degree of wettability; this determination was measured using a DSA 100 Krüss GmbH, German goniometer from Hamburg, using the sessile drop method with water. The samples were split, flattened, using glass plates, and dried at 40 °C in a vacuum oven for 4 h. For the determination, a drop of distilled water was deposited on the flat surface, and the angle formed between the surface and the liquid was measured. All of the measurements were carried out six times.

### 2.6. pH-Responsiveness

To determine the pH response of the samples, phosphate buffer solutions of pH 2, 3, 4, 5, 6, 8, 10, and 12 were prepared. A dry sample was weighed, and the solution with pH 2 was added, maintaining a controlled temperature at 25 °C for 2 h. Later the sample was removed, and the swelling percentage was calculated. The same procedure was used with the other solutions.

### 2.7. Load and Realese of Ciprofloxacin

#### 2.7.1. Ciprofloxacin Load

Samples around 100 mg were placed in vials with 3 mL of 0.012 µg/mL ciprofloxacin aqueous solution, at 25 °C, for 30 h. The loading time was determined by measuring the absorbance at different time intervals to assess the loading progress (4, 6, 8, 24, and 30 h). The amount of drug loaded was determined by measuring the difference in absorbance between a 0.012 µg/g ciprofloxacin solution without material and the solution with the material at each time interval at a wavelength of 266 nm, using a calibration curve. A SPECORD 200 PLUS brand spectrophotometer from Analytikjena (Germany) was used for the test. After that time, the samples were extracted and gently washed with distilled water. The calibration curve is shown in Supplementary Materials, in Figure S1.

#### 2.7.2. Ciprofloxacin Release

The samples loaded with ciprofloxacin were deposited in vials containing 3 mL of phosphate buffer solution at pH 7.4 and 37 °C with constant stirring (130 rpm). The cumulative release was monitored by taking measurements at 0.25, 0.5, 1, 2, 4, 6, 8, 24, 30, and 48 h at 266 nm using a UV–Vis spectrophotometer. The calibration curve is shown in Supplementary Materials, Figure S2. The release profiles were analyzed by the software DDSolver, and the detailed results are presented in Supplementary Materials.

### 2.8. Protein Adsorption Test

Approximately 80 mg of a sample were placed in PBS buffer solution at 37 °C for 2 h, then the sample was extracted and incubated in bovine serum albumin (BSA) protein solution at a 30 mg/mL concentration in PBS at 37 °C for 2 h. After this time, the materials were washed three times with PBS, and the adhered protein was extracted for later quantification.

For the extraction, 600 µL of 1% wt. SDS solution were added to the sample, previously incubated in BSA, and proceeded to be shaken at 130 rpm for 20 min and sonicated for 10 min, and finally vortexed for 30 s; this process was repeated 3 times. Finally, the protein concentration was quantified using the bicinchoninic acid method: 1 mL of the extraction solution was placed in a glass vial, 2 mL of the working solution (Table S1) was added, the mixture was gently shaken and heated at 60 °C for 30 min. After this time, the sample was cooled to room temperature, and the absorbance was measured using UV–Vis spectroscopy at 556 nm. Quantification was performed using a calibration curve (Supplementary Materials, Figure S3).

### 2.9. Cell Viability Assay

Cell viability was studied by observing the growth of BALB/3T3 murine embryonic fibroblasts (ATCC CCL-163, Manassas, VA, USA) in the presence of the modified materials. The assays were performed in 96-well plates seeded with 4 × 10^4^ cells/mL, using Dulbecco’s modified Eagle’s medium (DMEM) with FBS (fetal bovine serum, 10% *v*/*v*), penicillin-streptomycin (1% *w*/*v*) and gentamicin (10 μg/mL), for 24 h, under standard culture conditions (a humidified atmosphere of 5% CO_2_, at 37 °C). Samples of approximately six µg were added to the cell culture and incubated for 24 h. After this, the samples and the medium were removed, and the MIT kit was used to quantify them, measuring the absorbance at 620 nm (Multiskan FC, Thermo Scientific). Cell viability was obtained by comparing cell growth in the presence of samples with that obtained by cultures without them. The assay was performed in triplicate.

### 2.10. Bacterial Inhibition Test

A solution of *Escherichia coli* (ATCC25922) of 0.5 MF (1.5 × 10^8^ cfu/mL) was prepared in peptone water (pH: 7.2) and 2 mL of the solution was placed in a test tube, which had been previously sterilized (121 °C, 15 min and pressure of 1.6 kg/cm^2^). The material to be analyzed was placed in the medium and incubated at 37 °C for 24 h. The material was removed from the culture medium, and the optical density was measured at 600 nm by spectroscopy to quantify the inhibition of growth by comparing the difference between it and a solution whose bacterial growth was not affected by any external material [29]; the experiments were carried out in triplicate.

### 2.11. Bacterial Adhetion Test

Samples of approximately 30 mg were incubated in 0.5 MF *E. coli* solution for 24 h. Once removed and gently washed with sterile water to remove non-adhering bacteria, the samples were placed in a glass tube with 1 mL of sterile water and taken to the vortex for 3 min [30,31]. After vortexing, a 0.1 mL aliquot was taken and diluted to 1 mL. An amount of 0.1 mL of this solution was removed and seeded using the inverted plate technique. The solution was left to incubate for 24 h, and the former colonies were counted [32].

## 3. Results

### 3.1. Synthesis of PVC-g-4VP/4VPPS

**Figure 1:**
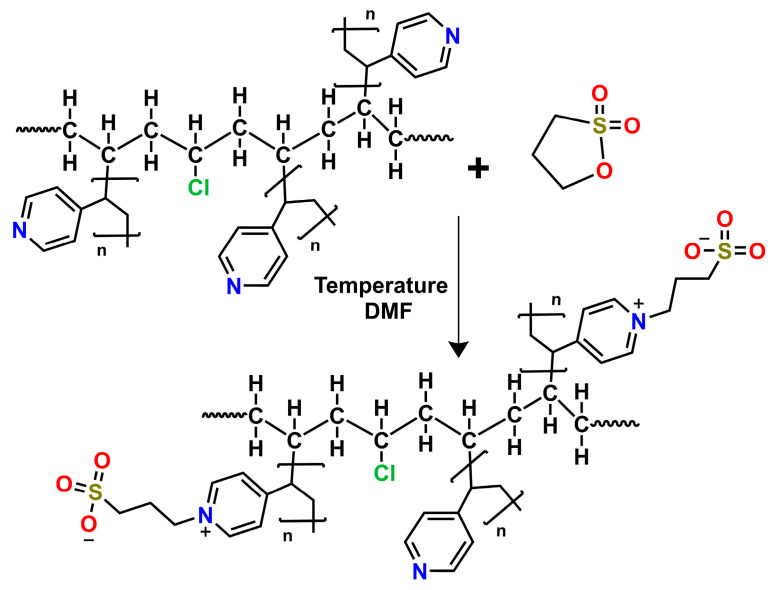
Reaction scheme for PVC-g-4VP/4VPSS.

Once the 12 and 23% 4VP grafts were obtained using the conditions reported in Duarte-Peña [33], we proceeded to functionalize them to form the PVC-*g*-4VP/4VPPS. One of the ways to obtain zwitterionic polymers is via the formation of the zwitterion after polymerization, that is, cationic or anionic polymers are functionalized with oppositely charged groups. This was the method used in the case of PVC-*g*-4VP/4VPPS materials, where 4VP was initially grafted onto the PVC matrix and later functionalized with PS to form the zwitterionic polymer 4-vinylpyridine propylsulfobetaine (4VPPS), resulting in a graft composed of 4VP/4VPPS. The process for functionalization consisted of a ring-opening alkylation reaction (Figure 1) [25].

**Figure 2:**
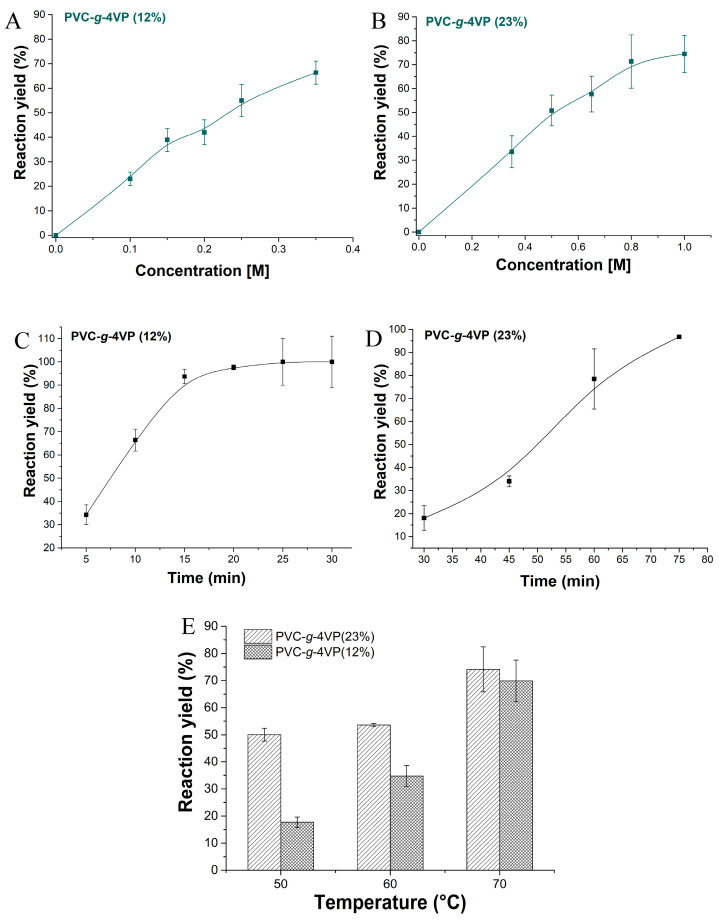
Effect of the reaction conditions in the yield percentage. (**A**) PS concentration effect for PVC-g-4VP(12%), conditions: 70 °C and 10 min, (**B**) PS concentration effect for PVC-g-4VP(23%), conditions: 70 °C and 75 min; (**C**) Time reaction effect for PVC-g-4VP(12%), conditions: 70 °C and 0.35 M PS; (**D**) Time reaction effect for PVC-g-4VP(23%), conditions: 70 °C and 0.8 M PS; and (**E**) Temperature effect, conditions for PVC-g-4VP(12%): 0.35 M PS and 10 min, and conditions for PVC-g-4VP(23%): 0.8 M PS and 75 min. Reported: mean ± standard error of the mean, *n* = 3.

Three factors were analyzed on the samples with 12 and 23% of 4VP to obtain the best reaction conditions: PS concentration, reaction time, and temperature. For this process, the reaction time was limited by the stability of the polymeric matrix in DMF, so the maximum reaction time used was 75 min. Figure 2A,B shows the behavior of the reaction yield when increasing the concentration of PS for samples with 12 and 23% of 4VP, respectively. In both cases, the reaction yield increased with a higher concentration; samples with 12% of 4VP required 0.35 M PS to reach 80 % of reaction yield, whereas the samples with 23% of 4VP reached these yields at a concentration of 0.8 and 1 M under the conditions used. Figure 2C,D show that the reaction yield increases with increasing reaction time, reaching yield values of 80 to 95% between 60 and 75 min for samples with 23% of 4VP and close to 100% at 20 min for 12% of 4VP.

On the other hand, the temperature played an important role during the reaction because this is an endothermic process that requires energy to overcome the activation barrier and shift the equilibrium towards the formation of the product. Three temperatures were tested: 50, 60, and 70 °C. Figure 2E shows the yields as a function of temperature for the two samples, the best yield for both was found at 70 °C.

Table 1 shows four graft ranges that were obtained from this synthesis. The material with grafts of 13% 4VP and 32% 4VPPS was discarded for characterization because there was a significant deformation of the catheter.

### 3.2. Infrared Spectroscopy and Themal Analysis

**Figure 3:**
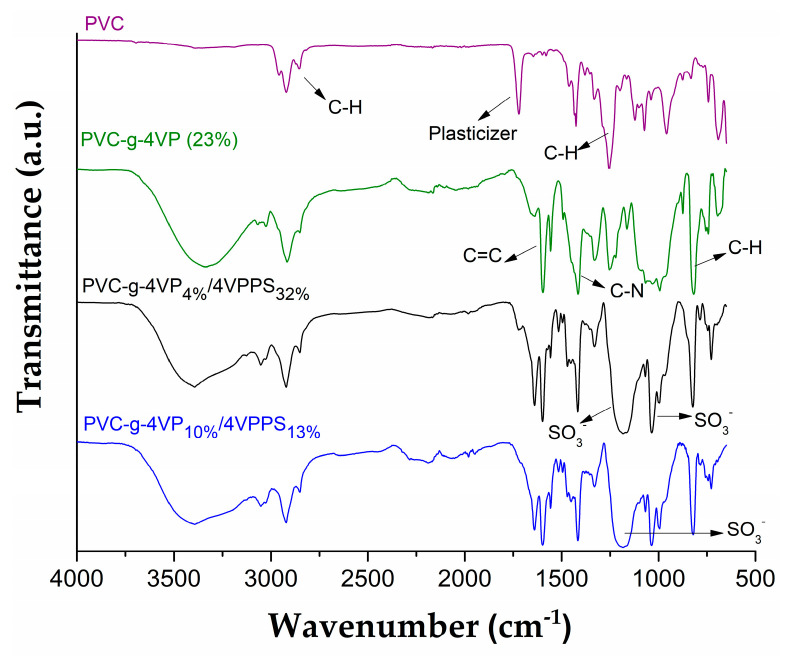
Infrared spectra of the neat and grafted PVC catheters using ATR module.

The PVC-*g*-4VP/4VPPS materials were characterized by infrared spectroscopy to determine the presence of the different functional groups, as shown in Figure 3. The control PVC catheter spectrum presented bands at 2992 cm^−1^ of C-H stretching of -CH_2_- groups, 1267 cm^−1^ of C-H flexions; additionally, bands at 1745 and 1459 cm^−1^ were presented, which are characteristics of the plasticizer. The PVC-*g*-4VP spectrum, apart from the previous bands, presented C=C stretching signals at 1597 and 1557 cm^−1^, and at 830 cm^−1^ of the C-H bends outside the plane of the aromatic amine [34,35]. Finally, the PVC-*g*-4VP/4VPPS spectra, together with the characteristic bands of the aromatic amine, show bands at 1039 and 1265 cm^−1^ corresponding to the symmetric and asymmetric stretching of the sulfonate group (SO_3_^−^), and the band 1680 cm^−1^ of the C=N stretches of the pyridine, corroborating the formation of the zwitterion 4VPPS [36].

**Figure 4:**
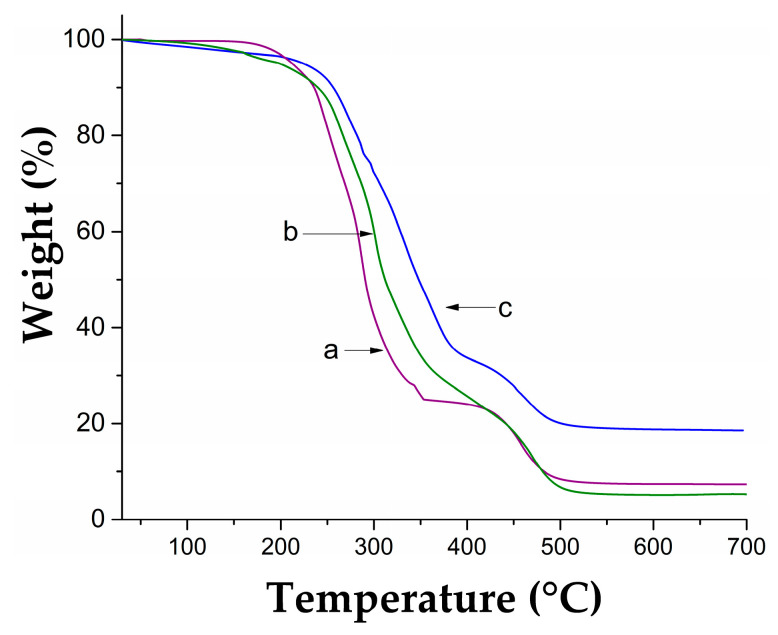
Thermograms: (**a**) PVC, (**b**) PVC-g-4VP, and (**c**) PVC-g-4VP/4VPPS.

TGA thermal analysis showed that the catheter PVC-*g*-4VP/4VPPS has greater thermal stability than PVC and PVC-*g*-4VP because it lost 10% weight at a temperature 18 °C higher (Table 2). PVC and PVC-*g*-4VP presented three decomposition temperatures: the first was attributed to the decomposition of the plasticizer, the second and more intense was produced by the dehydrochlorination of PVC, and the last was attributed to the total chain decomposition; the materials modified with 4VP presented an increase of around 20 degrees at all decomposition temperatures. Finally, the PVC-*g*-4VP/4VPPS catheters showed four decomposition temperatures: the first was at 272 °C due to the loss of the plasticizer, the second was at 333 °C due to the dehydrochlorination of PVC [37], the third was at 364 °C, which is consistent with the decomposition of the quaternary amine [38], and the last was at 453 °C, which corresponds to the carbon chain decomposition. Figure 4 shows the thermograms of each sample.

### 3.3. Swelling and Contact Angle

**Figure 5:**
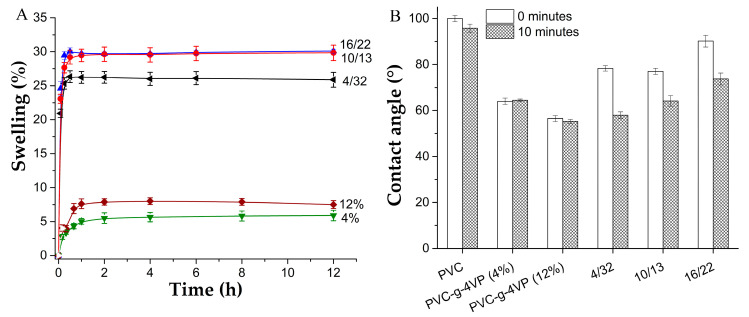
(**A**) Swelling curves in water at 25 °C, reported: mean ± standard error of the mean, *n* = 3. and (**B**) Water contact angle, reported: mean ± standard error of the mean, *n* = 6. PVC-*g*-4VP_4%_/4VPSS_32%_ (4/32), PVC-*g*-4VP_10%_/4VPSS_13%_ (10/13), and PVC-*g*-4VP_16%_/4VPSS_22%_ (16/22).

Figure 5A shows the swelling curves in water at 25 °C; PVC has a hydrophobic character for what it did not swell. Samples modified only with 4VP showed their maximum swell at 2 h. On the other hand, the catheters with 4VP/4VPPS reached their maximum swelling at 30 min and presented maximum percentages three times those reached by the samples without functionalization. Figure 5B shows the contact angles for the different materials; the PVC surface showed a contact angle of approximately 100°, which corresponds to its hydrophobic character; the modified materials, on the other hand, presented contact angles of less than 90°, indicating that the surface acquired hydrophilicity. The surfaces modified with 4VP/4VPPS initially had higher contact angles than those modified only with 4VP; however, after 10 min of water-surface interaction, the contact angles decreased to similar values.

### 3.4. pH-Sensitivity

**Figure 6:**
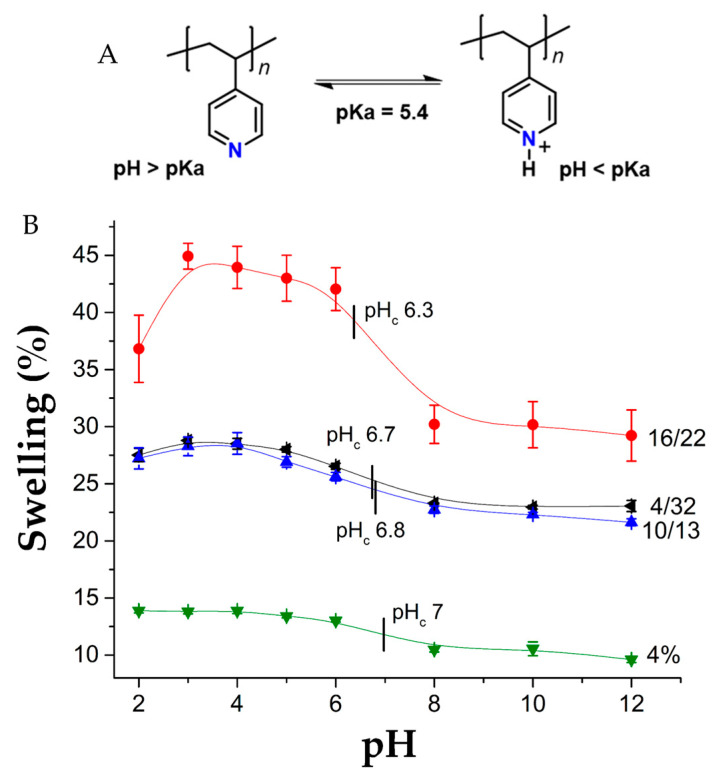
(**A**) Acid-base balance of 4VP and (**B**) pH-sensitivity, reported: mean ± standard error of the mean, *n* = 3.

4VP is a pH-sensitive polymer because it has an amino group in its structure that presents an acid-base balance (Figure 6A), with a pK_a_ around 5.4. At pHs below its pK_a_, the polymer is in its ionic form, so the chains suffer repulsion between them and swelling is greater. Figure 6B shows the behavior of the swelling as a function of the pH for the pristine PVC, PVC-*g*-4VP, and PVC-*g*-4VP/4VPPS. PVC did not present a pH response, whereas all the materials modified with 4VP showed this capability, with a critical pH in the range of 6.3 to 7.

### 3.5. Protein Adsortion Test

**Figure 7:**
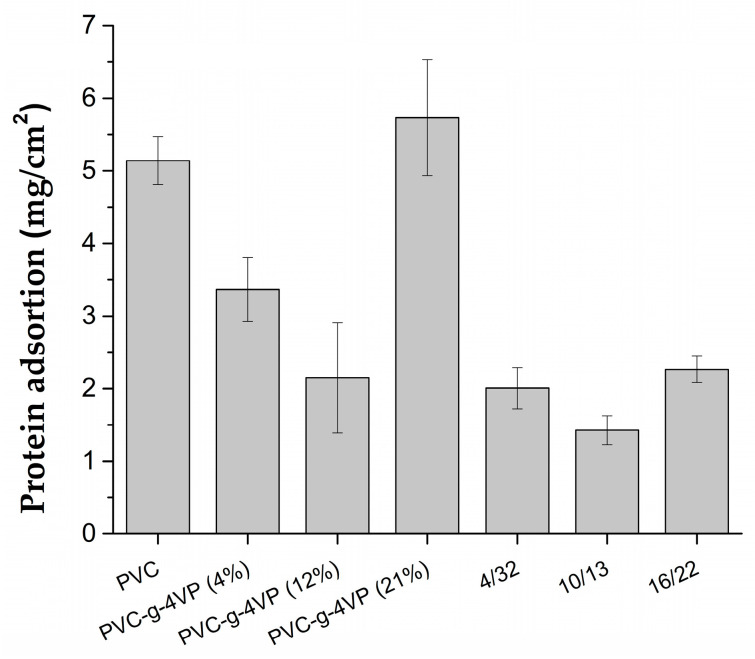
Protein adsorption on the polymer surface of different composition (incubation: 30% BSA solution in PBS, 37 °C, 2 h). Reported: mean ± standard error of the mean, *n* = 3.

The presence of the 4VPPS zwitterionic polymer decreased the percentage of protein adsorbed on the surface. Figure 7 shows the results; the material modified with 10% 4VP and 13% 4VPPS was the one that showed the highest antifouling capacity, reducing BSA adsorption by 74% compared to the unmodified PVC surface. This shows that the functionalization of the grafted 4VP was successful and the 4VPPS formed expresses its antifouling characteristics.

### 3.6. Load and Release of Ciprofloxacin

**Figure 8:**
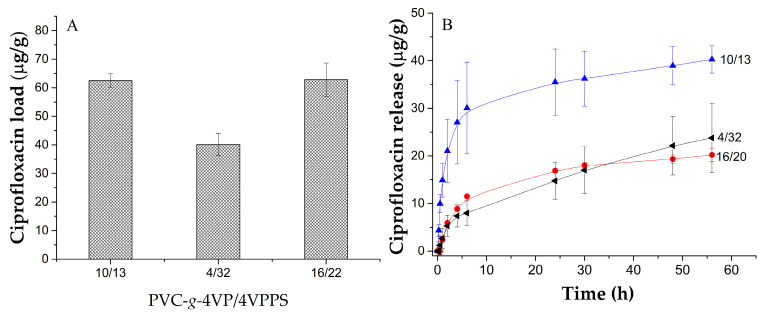
(**A**) Ciprofloxacin load (condition: 0.012 µg/mL, 25 °C por 30 h) and (**B**) Ciprofloxacin release profiles (condition: PBS a 37 °C). Reported: mean ± standard error of the mean, *n* = 3.

Ciprofloxacin loading and release assays were performed on PVC, PVC-*g*-4VP(4%), PVC-*g*-4VP_4%_/4VPPS_32%_, PVC-*g*-4VP_10%_/4VPPS_13%_, and PVC-*g*-4VP_16%_/4VPSS_22%_ samples. PVC and PVC-*g*-4VP(4%) samples did not show ciprofloxacin loading capacity, contrary to the samples modified with the zwitterionic polymer. Figure 8A shows the ciprofloxacin load for the three zwitterionic graft samples; in all the cases, the load reaches maximum levels after 30 h of interaction, but the amount of ciprofloxacin loaded by the PVC-*g*-4VP_10%_/4VPSS_13%_ and PVC-*g*-4VP_16%_/4VPSS_22%_ catheters was higher. Figure S4 shows the load profiles. However, release profiles (Figure 8B), showed that the sample PVC-*g*-4VP_10%_/4VPPS_13%_ had a final release of approximately 40 µg/mL, and the samples PVC-*g*-4VP_4%_/4VPPS_32%_ and PVC-*g*-4VP_16%_/4VPPS_22%_ released around 20 µg/mL; in all cases, the release was gradual until 30 h.

The release profiles were processed using DDSolver Excel software to determine the fitting model (details of the fit are specified in Supplementary Materials, Table S2). In all cases, following the values of the Akaike Information Criterion (AIC), the Model Selection Criterion (MSC), and the adjustment of squares (r^2^), the model that presented the highest affinity with the behavior of the materials was the Peppas–Sahlin model [39].

The Peppas–Sahlin model describes systems that present the contribution of two mechanisms (diffusional and relaxational) in the release process. This model is described by Equation (4), where M_∞_ is the amount of drug in an equilibrium state, M_t_ is the amount of drug released in the determined time, k_1_ represents the Fickian diffusion contribution, k_2_ the polymer chains relaxation contribution, and m is the Fickian diffusion exponent [40].

M_t_/M_∞_ = k_1_t^m^ + k_2_t^2m^ (4)

Table 3 shows the values of the Peppas–Sahlin model parameters for the different materials; all materials presented values of k_1_ that predominate over k_2_, indicating that the principal release mechanism is diffusion. However, for the material, the contribution of the chain relaxation mechanism is higher, possibly due to the greater amount of the zwitterionic polymer. The PVC-*g*-4VP_10%_/4VPPS_13%_ catheter released the highest amount of ciprofloxacin, with a release rate of approximately 60%.

The PVC-*g*-4VP_10%_/4VPPS_13%_ materials presented the best properties and fast synthesis conditions (0.35 M, 70 °C, 5 min, and 12% 4VP), which is why they were chosen to carry out cell viability, antimicrobial activity, and antifouling capacity tests.

### 3.7. Cell Viability

**Figure 9:**
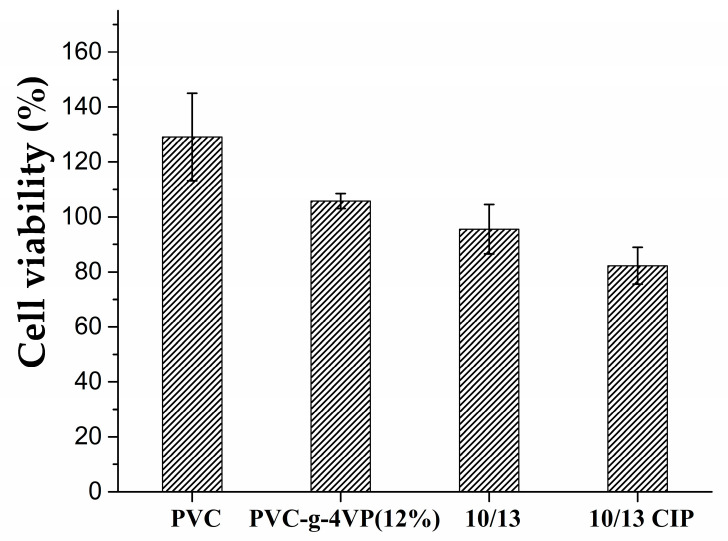
Cell viability result using BALB/3T3 murine embryonic fibroblasts and MIT assay. Reported: mean ± standard error of the mean, *n* = 3.

Figure 9 shows the results of cell viability using BALB/3T3 murine embryonic fibroblasts; it was observed that the control PVC increased cell growth, in the same way as the material modified with 4% 4VP. The material grafted with 4VP/4VPPS showed a decrease in cell growth of approximately 5%; when the material was loaded with ciprofloxacin, the cell growth decreased by 18%; however, the material can be considered non-cytotoxic according to ISO-10993-5-2009 [41].

### 3.8. Antimicrobial Activity and Antifouling Capability

**Figure 10:**
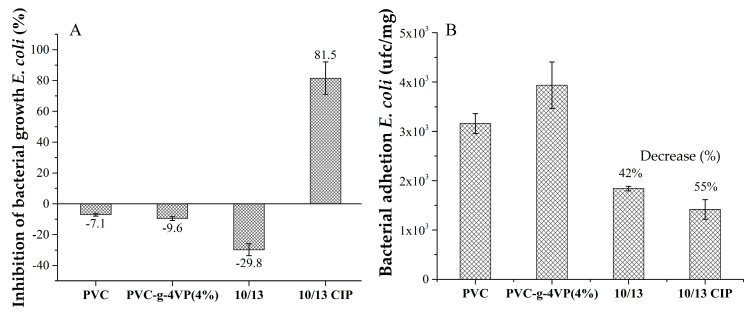
(**A**) Inhibition of bacterial growth for *E. coli* and (**B**) Bacterial adhesion of *E. coli.* Reported: mean ± standard error of the mean, *n* = 3.

Figure 10A presents the antibacterial capacity of the material based on its ability to inhibit the growth of *E. coli*. It was observed that PVC, PVC-*g*-4VP(4%), and PVC-*g*-4VP_10%_/4VPPS_13%_ do not have antimicrobial activity, on the contrary, PVC-*g*-4VP_10%_/4VPPS_13%_ loaded with ciprofloxacin achieved 82% inhibition at 24 h. On the other hand, Figure 10B presents the antifouling capacity of the material determined by the number of colony-forming units of *E. coli* bacteria, which adhered to the material when it was in direct contact with the *E. coli* solution of 1 × 10^8^ cfu for 24 h. It is observed that the material with binary graft presents a 42% decrease in bacterial adhesion in the control PVC and this effect is enhanced when loading ciprofloxacin, reaching a 55% decrease.

## 4. Discussion

Ionic polymer functionalization using oppositely charged ions is one of the ways to synthesize zwitterionic polymers. In this case, the functionalization of 4VP grafted onto PVC catheters made it possible to obtain the characteristics of the 4VPPS zwitterion while maintaining the pH-sensitivity of the 4VP system. The 4VP is a cationic polymer that reacts with PS to produce 4VPPS, by a ring-opening alkylation reaction (Figure 1). This form of zwitterion synthesis resulted in different percentages of grafting, which was impossible with a direct grafting method, that is, directly grafting the 4VPPS monomer. The difficulty of working with the 4VPPS monomer was due to its insolubility in most solvents; a 1 M saline solution is required to solubilize it [42,43]. The formation of grafts using ionizing radiation represents an advantageous method for medical device modification because the obtained materials do not present contamination by agents used for the polymerization [44,45].

The poly (4VPPS) is an aromatic zwitterionic polymer that has demonstrated antifouling capabilities in biomedicine, electronics [46], and water treatment. Venault, et. al. synthesized 4VPPS hydrogels to be used as medical device coatings. They demonstrated that 4VPPS coatings present a bio-antifouling capability similar to that of sulfobetaine methacrylate (SBMA), one of the most common zwitterionic polymers, employed to produce antifouling surfaces by coating, grafting, or copolymerization [47,48,49,50]. Additionally, 4VPPS coatings were more resistant to temperature than SBMA coatings because they retained their bio-antifouling properties after being heated at 121 °C for 1 h (sterilization conditions), representing an advantage in the biomedical field, where most of the devices require sterilization [25]. On the other hand, Gui, et. al. copolymerized acrylamide with 4VPPS to produce a zwitterionic poly-acrylamide that showed thermic stability and good properties as a flocculant of cationic and anionic inorganic contaminants due to its electrostatic characteristics [51].

Materials grafting with 4VPPS showed the ability to load and release ciprofloxacin due to the hydrophilicity and pH-sensitivity of the system. In addition, the modification provided the material with the antifouling property of zwitterionic polymers, allowing a significant decrease in both the adhesion of the BSA protein and *E. coli*. A synergistic effect was observed when loading the material with ciprofloxacin, increasing its capacity to prevent the adhesion of *E. coli* by 13%, for the PVC-*g*-4VP_10%_/4VPPS_13%_ catheter. The materials were also shown to be non-toxic since they did not significantly affect cell viability. The modification of the catheters allows the creation of antimicrobial systems with the capacity to release an antibiotic at the site of interest, making it a localized drug delivery system, increasing its effectiveness and decreasing the side effects.

## 5. Conclusions

The functionalization of the 4VP grafted onto PVC catheters was successful and obtained materials with different percentages of 4VP and 4VPPS in their composition. Although all the modified catheters showed hydrophilicity and pH sensitivity, the PVC-*g*-4VP_10%_/4VPPS_13%_ catheter was the one that showed the best capacities for loading and releasing ciprofloxacin, achieving an inhibition of *E. coli* growth of approximately 82% in 24 h. In addition, due to the presence of zwitterion, this material showed antifouling characteristics, which decreased the adhesion of BSA by 74% and the adhesion of *E. coli* by 55%. This material is a pH-sensitive system with dual antimicrobial capacity and is therefore a promising alternative for developing devices with less tendency towards biocontamination.

## Figures and Tables

**Table 1:** Materials synthesized.

Samples
-◄- PVC-*g*-4VP_4%_/4VPPS_32%_
-▲- PVC-*g*-4VP_10%_/4VPPS_13%_ -●- PVC-*g*-4VP_16%_/4VPPS_22%_ PVC-*g*-4VP_13%_/4VPPS_32%_

**Table 2:** Thermogravimetric analysis.

Sample	10% Weight Loss (°C)	Decomposition Temperatures (°C)
PVC	236	243 287 457
PVC-*g*-4VP	240	261 301 470
PVC-*g*-4VP/4VPPS	256	272 333 364 453

**Table 3:** Peppas–Sahlin model parameters.

Parameter	PVC-*g*-4VP_4%_/4VPPS_32%_	PVC-*g*-4VP_10%_/4VPPS_13%_	PVC-*g*-4VP_16%_/4VPPS_22%_
k_1_	13.22	14.48	13.63
k_2_	0.523	−0.678	−0.657
m	0.573	0.377	0.601

## Data Availability

Not applicable.

## Supplementary Materials

The following supporting information can be downloaded at: https://www.mdpi.com/article/10.3390/pharmaceutics15030960/s1, Figure S1: Calibration curves to quantify load of ciprofloxacin; Figure S2. Calibration curves to quantify release of ciprofloxacin; Figure S3. Calibration curve to BSA quantification (Abs 556 nm); Figure S4. Profiles of ciprofloxacin load; Table S1: Working solution preparation; Table S2. Parameters to select the drug release model.
