# Supplementary material for: Catheters with Dual-Antimicrobial Properties by Gamma Radiation-Induced Grafting
Source: Pharmaceutics. 2023 Mar 16;15(3):960. doi: 10.3390/pharmaceutics15030960 (PMC10056229; doi:10.3390/pharmaceutics15030960)
Supplement: Supplementary file 1 [file pharmaceutics-15-00960-s001.zip › pharmaceutics-2252344-supplementary.pdf]

pharmaceutics

Supplementary material

Catheters with dual-antimicrobial properties by gamma radiation-induced grafting

Lorena Duarte-Peña [1,*], Héctor Magaña [2] and Emilio Bucio [1,*]

[1] Departamento de Química de Radiaciones y Radioquímica, Instituto de Ciencias Nucleares, Universidad Nacional Autónoma de México, Circuito Exterior, Ciudad Universitaria, Ciudad de Mexico 04510, Mexico
[2] Facultad de Ciencias Químicas e Ingeniería, Universidad Autónoma de Baja California, Calzada Universidad 14418, Parque Industrial Internacional Tijuana, Tijuana 22390, Mexico
* Correspondence: lorena.duarte@correo.nucleares.unam.mx (L.D.-P.); ebucio@nucleares.unam.mx (E.B.)

Citation: Duarte-Peña, L.; Magaña, H.; Bucio, E. Catheters with Dual-Antimicrobial Properties by Gamma Radiation-Induced Grafting. *Pharmaceutics* **2023**, *15*, 960. https://doi.org/10.3390/pharmaceutics15030960

Academic Editor: Ana Isabel Fernandes

Received: 14 February 2023
Revised: 10 March 2023
Accepted: 14 March 2023
Published: date

Copyright: © 2023 by the authors. Submitted for possible open access publication under the terms and conditions of the Creative Commons Attribution (CC BY) license (https://creativecommons.org/licenses/by/4.0/).

1. Calibration curve of ciprofloxacin load

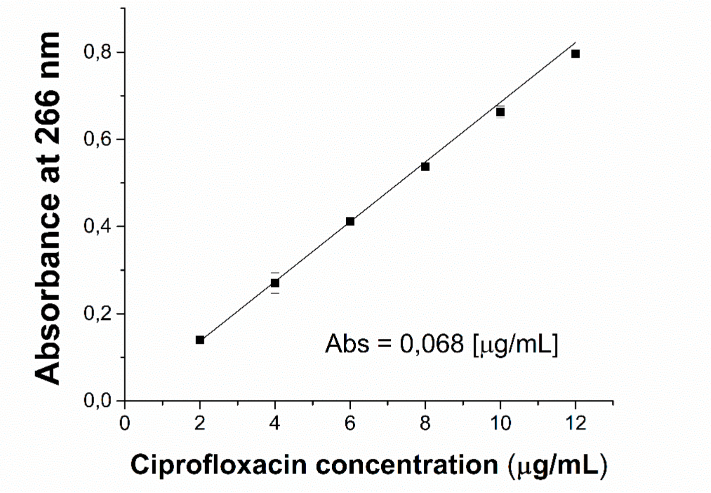

Figure S1. Calibration curves to quantify load of ciprofloxacin

2. Calibration curve of ciprofloxacin release

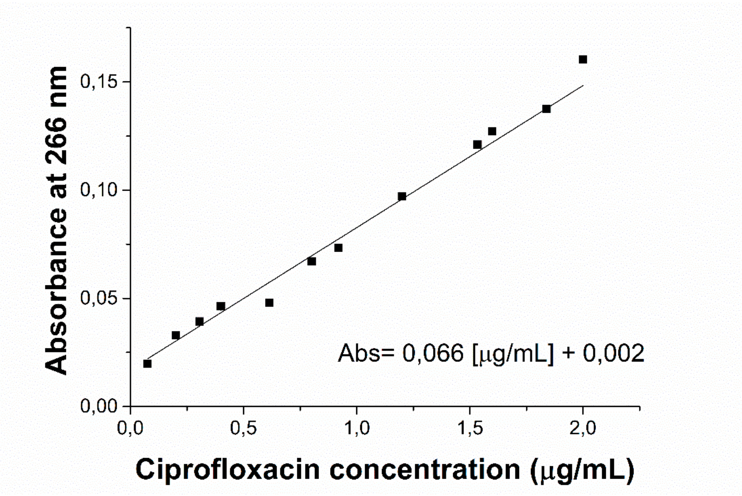

Figure S2. Calibration curves to quantify release of ciprofloxacin

3. BSA quantification: working solution preparation

The working solution is a light green hue solution that consists of 50 parts of solution A and 1 part of solution B, Table S1 shows the grams of substances used for each solution.

Table S1. Working solution preparation

**Solution A (100 mL at pH 11.25 adjusted with NaOH)**	**Solution B (100 mL)**
0.1 g sodium bicarbonate<br>2.0 g sodium carbonate dihydrate<br>0.16 g sodium tartrate (dihydrate)<br>0.4 g sodium hydroxide<br>0.95 g sodium bicarbonate	4 g copper sulfate (II) pentahydrate

4. BSA quantification: calibration curve

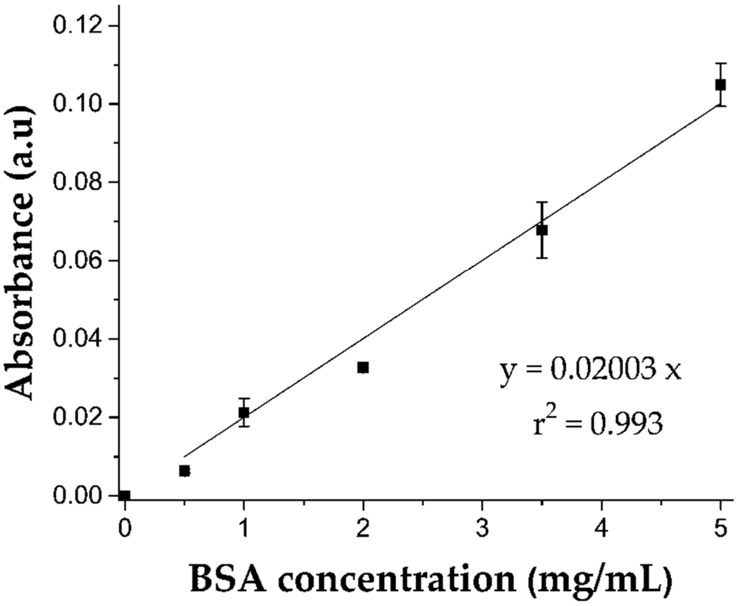

Figure S3. Calibration curve to BSA quantification (Abs 556 nm).

5. Parameters to select the drug release model

Table S2. Parameters to select the drug release model

Parameter	PVC-g-4VP$_{4\%}$/4VPPS$_{32\%}$	PVC-g-4VP$_{10\%}$/4VPPS$_{13\%}$	PVC-g-4VP$_{16\%}$/4VPPS$_{22\%}$
		Higuchi Model	
R^2	0.982	0.980	0.963
AIC	57.96	80.47	71.98
MSC	3.003	0.779	2.156
		Korsmeyer-Peppas Model	
R^2	0.986	0.994	0.975
AIC	56.68	56.79	70.34
MSC	3.121	2.380	2.304
		Peppas-Sahlin Model	
R^2	0.994	0.996	0.992
AIC	52.59	56.72	64.04
MSC	3.493	2.937	2.877

6. Profiles of ciprofloxacin load

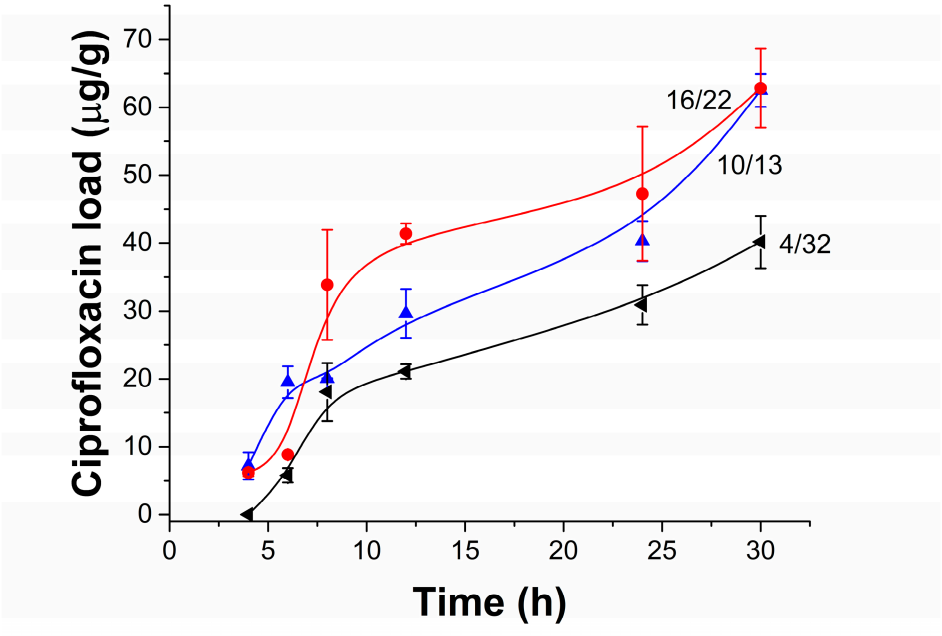

Figure S4. Profiles of ciprofloxacin load
